# Loss of RNA-binding protein CELF2 promotes acute leukemia development via FAT10-mTORC1

**DOI:** 10.1038/s41388-024-03006-3

**Published:** 2024-03-21

**Authors:** Tengxiao Guo, Yuxia Wang, Xiaolu Sun, Shuaibing Hou, Yanjie Lan, Shengnan Yuan, Shuang Yang, Fei Zhao, Yajing Chu, Yuanwu Ma, Tao Cheng, Jia Yu, Bing Liu, Weiping Yuan, Xiaomin Wang

**Affiliations:** 1 State Key Laboratory of Experimental Hematology, National Clinical Research Center for Blood Diseases, Haihe Laboratory of Cell Ecosystem, Institute of Hematology & Blood Diseases Hospital, Chinese Academy of Medical Sciences & Peking Union Medical College, Tianjin, 300020 China; 2 Biomedical Center of Qingdao University, Qingdao, 266000 China; 3 Tianjin Institutes of Health Science, Tianjin, 301600 China; 4 Key Laboratory of Human Disease Comparative Medicine, National Health Commission of China (NHC), Institute of Laboratory Animal Science, Peking Union Medicine College, Chinese Academy of Medical Sciences, Beijing, 100021 China; 5 Department of Biochemistry, Institute of Basic Medical Sciences, Chinese Academy of Medical Sciences (CAMS) & Peking Union Medical College (PUMC), Beijing, 100005 China; 6 State Key Laboratory of Proteomics, Translational Medicine Center of Stem Cells, 307-Ivy Translational Medicine Center, Laboratory of Oncology, Affiliated Hospital, Academy of Military Medical Sciences, Beijing, 100071 China; 7 Key Laboratory of Carcinogenesis and Translational Research (Ministry of Education/Beijing), Department of Lymphoma, Peking University Cancer Hospital & Institute, Beijing, 100142 China; 8 State Key Laboratory of Experimental Hematology, Department of Stem Cell and Regenerative Medicine, Institute of Hematology & Blood Diseases Hospital, Chinese Academy of Medical Sciences & Peking Union Medical College, Tianjin, 300020 China

**Keywords:** Cancer stem cells, Acute myeloid leukaemia, Cancer models

## Abstract

RNA-binding proteins (RBPs) are critical regulators for RNA transcription and translation. As a key member of RBPs, ELAV-like family protein 2 (CELF2) has been shown to regulate RNA splicing and embryonic hematopoietic development and was frequently seen dysregulated in acute myeloid leukemia (AML). However, the functional role(s) of CELF2 in hematopoiesis and leukemogenesis has not been fully elucidated. In the current study, we showed that *Celf2* deficiency in hematopoietic system led to enhanced HSCs self-renewal and differentiation toward myeloid cells in mice. Loss of *Celf2* accelerated myeloid cell transformation and AML development in MLL-AF9-induced AML murine models. Gene expression profiling integrated with RNA immunoprecipitation sequencing (RIP-Seq), together with biochemical experiments revealed that CELF2 deficiency stabilizes *FAT10* mRNA, promotes FAT10 translation, thereby increases AKT phosphorylation and mTORC1 signaling pathway activation. Notably, combination therapy with a mTORC1 inhibitor (Rapamycin) and a MA9/DOTL1 inhibitor (EPZ-5676) reduced the leukemia burden in MLL-AF9 mice lacking *Celf2* in vivo. Our study elucidated a novel mechanism by which the CELF2/FAT10-AKT/mTORC1 axis regulates the proliferation of normal blood cells and the development of AML, thus providing potential therapeutic targets for myeloid leukemia suppression.

## Introduction

Acute myeloid leukemia (AML) is an aggressive hematological malignancy characterized by uncontrolled expansion of poorly differentiated myeloid cells caused by various genetic and epigenetic changes in hematopoietic cells [1]. Despite of an increasing understanding of the molecular basis of AML pathogenesis, the overall survival of adult patients has only improved modestly over the past 30 years [2], highlighting the need to identify better therapeutic targets and develop more effective strategies for AML treatment. Accumulated evidence revealed that the perturbations in RNA-binding proteins (RBPs)-mRNA networks were causally associated with hematological malignancies, including leukemia [3].

RBPs are proteins that can bind single or double-stranded RNA and thereby participate in forming ribonucleoprotein (RNP) complexes to affect the fate of mRNA. As post-transcriptional regulators, they participated in many aspects of RNA post-transcriptional modifications such as RNA splicing, polyadenylation, stability, localization, translation, and degradation [4]. RBPs provide the abundance and diversity of the mRNA/protein that contribute to cell fate decisions [5]. The dysregulation of RBPs expression has been shown to affect the progression of myeloid malignancies [3–5]. For example, increased expression of *MSI2* enhanced the translation of a number of critical genes thereby acerbate the aggressiveness of leukemia [6–8]. The increase of protein expression of IGF2BP2 promoted the leukemia cell proliferation by stabilizing the mRNA of ALDH1A1 and HOXB4 [9, 10]. Although the alterations of RBPs are thought to be important and of therapeutic potential, only limited RNA regulators have been functionally studied in hematological malignancies. Thus, deciphering the regulatory networks and mechanisms of RBPs will provide a better understanding of leukemia cell biology, as well as to unveil novel therapeutic targets.

CELF2 (CUG binding protein, elav‐like family member 2) is a canonical RNA-binding protein which regulates mRNA splicing and stabilization to ensure cellular homeostasis [11–14]. *CELF2* gene is located on chromosome 10p, a region frequently lost in human cancers. Studies have shown that RBP CELF2 is involved in tumorigenesis. The expression of CELF2 was down-regulated in ovarian cancer cells, and led to over-proliferation, migration, and invasion of ovarian cancer cells in vitro and in vivo [15]. The lower expression of CELF2 was correlated with high chemo-resistance and early dissemination of pancreatic cancer through the COX-2-mediated cyto-protective pathways [16]. Interestingly, CELF2 is shown to be highly expressed in many hematopoietic organs and is essential for the transition of endothelial cells to T1 pre-HSCs [17]. ICGC (International Cancer Genome Consortium) Data Portal showed that *CELF2* was frequently mutated in leukemia (https://dcc.icgc.org/genes/ENSG00000048740/mutations), suggesting a potential role of CELF2 in leukemogenesis. However, the functional roles of CELF2 in the hematopoiesis and leukemogenesis are not fully investigated.

In the current study, we aimed to functionally dissect the role of CELF2 in leukemia using *Celf2*-deficient MLL-AF9 mouse models. We found that loss of CELF2 promoted myeloid leukemia development by upregulating FAT10-mTORC1 pathway, suggesting a potential tumor suppressing role for CELF2.

## Results

### CELF2 deletion is associated with myeloid leukemia transformation and enhances HSCs self-renewal

To investigate the potential role of CELF2 in leukemia, we first analyzed the Pediatric Acute Myeloid Leukemia dataset from the Cancer Genome Atlas (TCGA) (http://www.cbioportal.org/). We found that *CELF2* deletion occurred in 9.4% of acute myeloid leukemia (AML) patients (Fig. S1A) and the survival time of AML patients with *CELF2* deletion was significantly shorter than that of the others (Fig. S1B). Interestingly, MLL related genetic alteration accompanied with CELF2 deletion accounted for 6.38% of all the 94 AML patients (Table S1). Additional mRNA expression analyses of *CELF2* showed the expression is highest in normal myeloid cells in peripheral blood (PB) cells using data from human leukemia databases (GSE42519 and GSE13159, http://servers.binf.ku.dk/bloodspot), while it decreased in different subtypes of AMLs (Fig. S1C). We further confirmed that *CELF2* mRNA level was significantly lower in primary AML cells from patients’ total bone marrow (BM) when compared with the mononuclear cells from healthy donor BM (Fig. S1D). These data suggested that CELF2 may play an important role in leukemogenesis in AML patients.

**Fig. 1:**
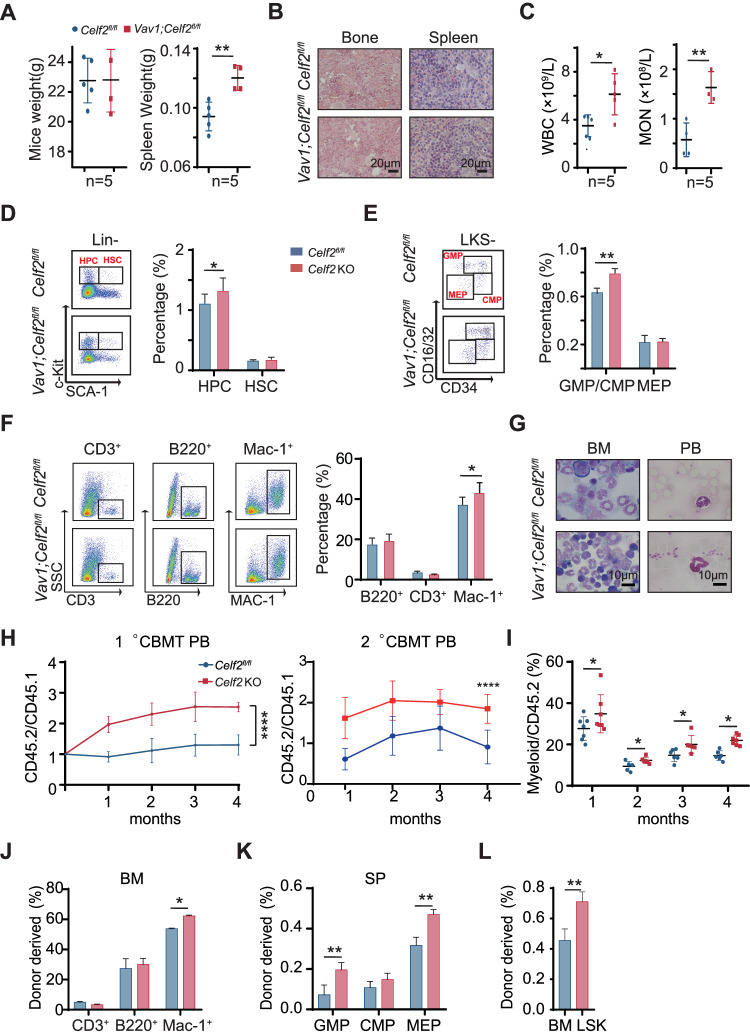
CELF2 deficiency is associated with AML and deletion of *Celf2* leads to mild expansion of myeloid cells in mice. **A** The weight of mice and the weight of spleen from 8 weeks *Celf2*^*fl/fl*^ or *Celf2* KO mice (*n* = 5). **B** H&E staining of BM and spleen in *Celf2*^*fl/fl*^ or *Celf2* KO mice at 8 weeks. **C** Blood count analysis in the PB of *Celf2*^*fl/fl*^ or *Celf2* KO mice at 8 weeks for white blood cells (WBC), monocytes (MON) (*n* = 5). **D** Representative flow cytometry plot and frequencies of HPCs and HSCs in the bone marrow of *Celf2*^*fl/fl*^ or *Celf2* KO mice at 8 weeks (*n* = 5). **E** Representative flow cytometry plot and frequencies of the GMPs, CMPs and MEPs in total BM cells of *Celf2*^*fl/fl*^ or *Celf2* KO mice at 8 weeks (*n* = 5). **F** Wright-Giemsa stain of peripheral blood and bone marrow in *Celf2*^*fl/fl*^ or *Celf2* KO mice at 8 weeks. **G** Percentages of different mature lineage cells in BM of *Celf2*^*fl/fl*^ or *Celf2* KO mice at 8 weeks (*n* = 5). **H** Total BM cells from *Celf2*^*fl/fl*^ or *Celf2* KO mice were transplanted into lethally irradiated recipient mice (CD45.1) together with equal numbers of CD45.1 competitor BM cells. Flow cytometry analysis of the contribution of *Celf2*^*fl/fl*^ (CD45.2^+^) and *Celf2* KO (CD45.2^+^) donor-derived cells in the PB of recipient mice after competitive BMT in primary transplantation assay (left panel) and secondary transplantation assay (right panel) (*n* = 7). **I** The percentages of *Celf2*^*fl/fl*^ and *Celf2* KO donor-derived myeloid cells in the PB of recipient mice after competitive BMT (*n* = 7). **J** Percentage of donor-derived different mature lineage cells compartments in the BM of recipients at 16 weeks after competitive BMT (*n* = 7). **K** Percentage of donor-derived GMPs, CMPs and MEPs compartments in spleen of recipients at 16 weeks after competitive BMT (*n* = 7). **L** Percentage of donor-derived LSK cells at 16 weeks after competitive BMT (*n* = 7). The data are presented as the mean ± SD and one-way ANOVA, **P < 0.01; ****P < 0.0001.

To further dissect the role of CELF2 in hematopoiesis and leukemogenesis, we generated *Celf2*^*fl*/*fl*^ mice (*Celf2* WT) (Fig. S1E) and crossed them with *Vav1-Cre* mouse line to generate hematopoietic-specific *Celf2-*deficient mice (*Vav1-Cre; Celf2*^*fl/fl*^, will be referred to *Celf2* KO) (Fig. S1F, G). We found that the weight of spleen was significantly increased in *Celf2* KO mice when compared with the controls, while the body weight of *Celf2* KO and control mice was similar (Fig. 1A). The histopathologic examination showed no dysplasia in bone marrow (BM) and spleen in *Celf2* KO mice (Fig. 1B). *Celf2* KO mice had higher white blood cell (WBC) counts and monocytes in PB than that of *Celf2* WT mice (Fig. 1C). Flow cytometric analysis of BM from two groups of mice showed that the percentage of HSCs was similar, whereas the percentage of HPCs and GMPs/CMPs was significantly increased in *Celf2* KO mice in comparison with the controls respectively (Fig. 1D, E). The absolute number of HPCs, HSCs, CMPs, CMPs and MEPs from CELF2 KO mice was equivalent to that of controls (Fig. S1H). Furthermore, we used SLAM markers to characterize the LT-HSCs and found the absolute number of LT-HSCs from CELF2 KO mice was significantly increased when compared with that of controls (Fig. S1I). Although the percentage of myeloid cells was higher in BM of *Celf2* KO mice than that of controls (Fig. 1F), the differentiation of myeloid cells was normal in PB and BM of *Celf2* KO mice by Wright-Giemsa staining assay (Fig. 1G). These results indicated that loss of *Celf2* increases the myeloid progenitor cells pool and leads to the expansion of myeloid cells mildly in vivo.

To further probe the potential role of Celf2 in regulating the function of HSCs, we performed competitive transplantation assay by transplanting BM cells from *Celf2* KO or *Celf2* WT mice together with CD45.1^+^ BM cells into lethally irradiated CD45.1^+^ recipient mice. We examined the frequency of reconstituted cells (CD45.2^+^) in PB every four weeks after transplantation and found that the chimerism of *Celf2* KO cells (CD45.2^+^) in PB was significantly higher than the controls (Fig. 1H, left panel). Additionally, in our serial competitive transplantation assay, while the chimerism of *Celf2* KO cells (CD45.2^+^) in PB was twice that of the control in primary (1°), the change was decreased in secondary (2°) transplantation (Fig. 1H, right panel), suggesting that the competitive advantage of *Celf2* KO HSCs may be reduced during serial transplantations. Notably, the myeloid cells derived from *Celf2* KO BM cells were increased (Fig. 1I). We also found that the percentage of T cells derived from *Celf2* KO BM cells were lower than that of controls (Fig. S1J), while the percentage of B cells derived from *Celf2* KO BM cells were higher than that of controls (Fig. S1K). The recipient mice were sacrificed at 4 months after transplantation, and the percentages of donor-derived *Celf2* KO CMPs/GMPs in spleen and myeloid cells in BM were found to be increased in recipient mice (Fig. 1J, K). The percentage of donor-derived *Celf2* KO CMPs/GMPs in BM was equivalent to that of controls (Fig. S1L). Notably, the number of donor-derived *Celf2* KO LSK^+^ cells was much higher in the BM of recipient mice after four-month transplantation, when compared with that of the controls (Fig. 1L). In summary, these results demonstrated that *Celf2* deletion enhances hematopoietic reconstitution but doesn’t lead to malignant hematopoiesis.

### *Celf2* deficiency lowers the threshold of malignant transformation of hematopoietic progenitor cells to leukemia

**Fig. 2:**
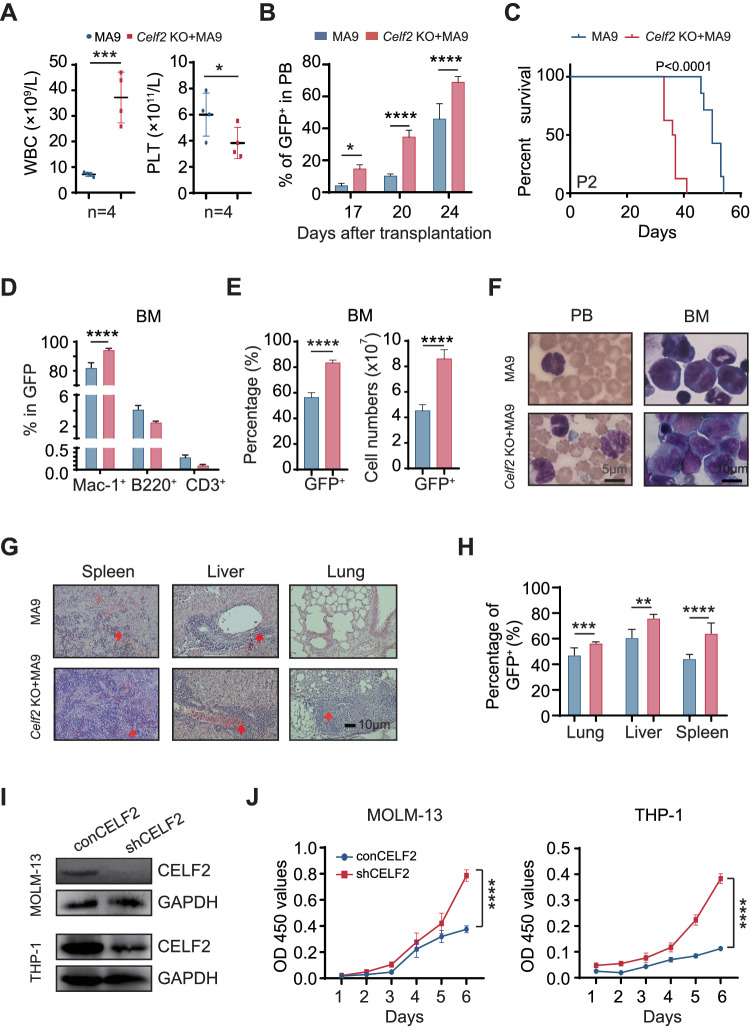
*Celf2* deficiency promotes disease progression in AML. **A** Complete blood count analysis of peripheral blood (PB), white blood cells (WBC) and platelets (PLT) in MA9 or *Celf2* KO + AML mice (*n* = 5). **B** The percentage of GFP^+^ cells in PB after transplantation (*n* = 7). **C** Kaplan–Meier survival analysis of MA9 or *Celf2* KO + MA9 mice in secondary transplant (*n* = 7). The data are presented as the mean ± SD and one-way ANOVA, **P < 0.01; ****P < 0.0001. **D** The lineage commitments of the transduced HSPCs were evaluated by examining the percentages of myeloid cells, B lymphocytes and T lymphocytes within the GFP^+^ cells. The data for the last time point were collected when the mice were euthanized (*n* = 5). **E** The percentage and absolute number of GFP^+^ cells in BM of MA9 or *Celf2* KO + AML mice (*n* = 5). **F** Wright-Giemsa stain of PB and BM. **G** H&E staining of spleen, liver and lung. **H** The percentage of GFP^+^ cells in spleen, liver and lung from recipient mice at end stage (*n* = 5). **I** Western blotting showing CELF2 KD efficiency in MOLM-13 and THP-1 leukemia cells after transduction with shRNA lentiviruses targeting *CELF2*. **J** Cell proliferation in the indicated cell lines after transduction with lentivirus expressing control or CELF2-specific shRNA. Panel **I**, **J** shows 1 representative experiment of at least 3 independent experiments.

In contrast to many other leukemia-associated mutations, MLL-fusions are powerful oncogenes that could transform both hematopoietic stem cells and committed progenitor cells. To evaluate the potential synergistic action of *Celf2* loss and MLL-fusion in the leukemia-initiating event in AML development, we transduced hematopoietic progenitor cells from *Celf2* KO and *Celf2* WT mice with retrovirus expressing MA9 (Fig. S2A and Supplemental method). In this model, *Celf2* KO + MA9 mice showed more aggressive leukemia phenotypes than *Celf2* WT + MA9 mice, including higher WBCs and lower PLT in PB (Fig. 2A). Moreover, the percentage of leukemia cells from 17 to 24 days after transplantation showed accelerated leukemia development in *Celf2* KO + MA9 mice (Fig. 2B), leading to markedly accelerated mortality in this group (*Celf2* KO + MA9 mice median survival = 36 days; MA9 mice median survival = 50 days; log-rank test *P* < 0.0001; Fig. 2C). Further analysis showed that the GFP^+^ leukemia cells in these two groups were mainly myeloid cells (Mac-1^+^) (Fig. 2D). The percentage and absolute number of GFP^+^ leukemia cells were significantly increased in BM of *Celf2* KO + MA9 mice, when compared with that of MA9 mice (Fig. 2E). Wright-Giemsa staining showed that the counts of abnormal leukemia cells was increased in PB and BM of *Celf2* KO + MA9 mice when compared with that of MA9 mice (Fig. 2F). The percentage of GFP^+^ leukemia cells and the degree of extramedullary infiltration were also increased in *Celf2* KO + MA9 mice than that in MA9 mice, including in the spleen, liver and lung (Fig. 2G, H).

To investigate whether the CELF2 deletion is functionally relevant to human myeloid leukemia development, we knocked down (KD) the CELF2 expression in MOLM-13 and THP-1 (both contain MLL-AF9 mutation) cell lines (Fig. 2I), and found that CELF2 KD increased the growth of MOLM-13 and THP-1 cells (Fig. 2J), decreased the apoptosis of MOLM-13 and THP-1 cells (Fig. S2B). When we overexpressed (OE) the CELF2 in MOLM-13 cells (Fig. S2C), we found that CELF2 OE significantly decreased the expression of P-AKT and inhibited the growth of MOLM-13 cells in vitro (Fig. S2C, D). Taken together, these results demonstrated that CELF2 loss could significantly accelerate myeloid leukemia growth in vitro and lower the threshold of MLL-AF9-induced AML development in vivo.

### *Celf2* deficiency potentiates the capability of leukemia stem cells in MA9-induced AML

**Fig. 3:**
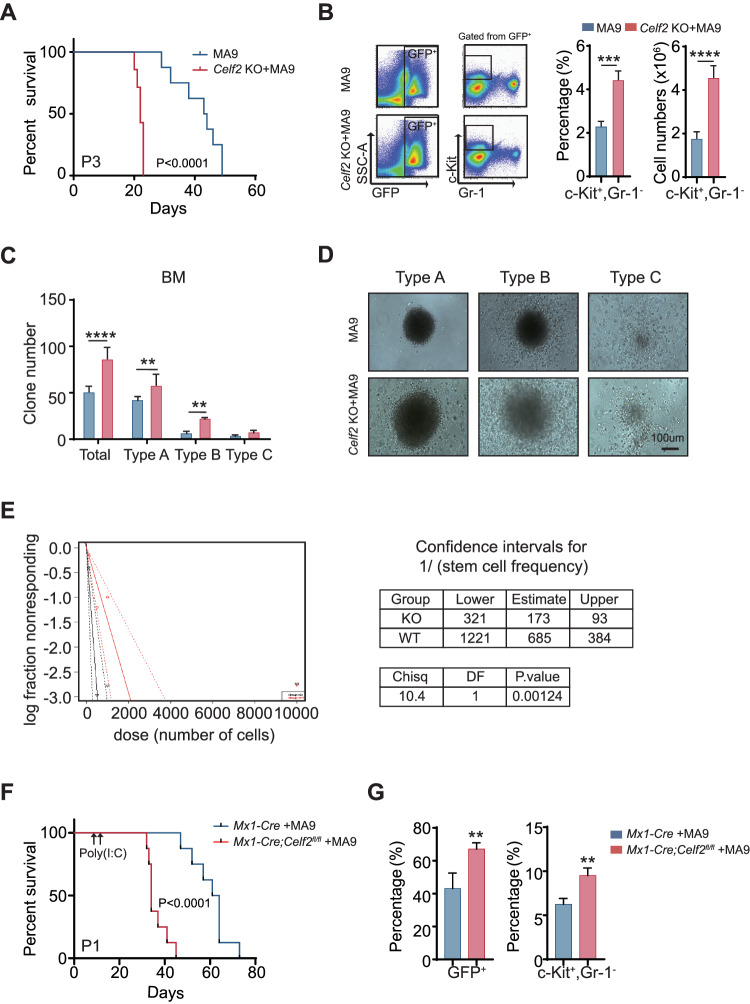
Deletion of *Celf2* leads to increases the number and capacity of LSCs. **A** Kaplan–Meier survival analysis of MA9 or *Celf2* KO + AML mice in tertiary transplant (*n* = 8). The data are presented as the mean ± SD and one-way ANOVA, **P < 0.01; ****P < 0.0001. **B** The percentage of c-Kit^+^Gr-1^−^ cells from BM in MA9 or *Celf2* KO + AML mice (*n* = 5). Colony formation assay (**C**), and representative colony images (**D**) of murine leukemia cells. Cells were cultured in MethoCult M3434 for 7 days. **E** The frequency of LSCs was determined by serial dilution and competitive transplantation assay. **F** Kaplan–Meier survival analysis of *Mx1-Cre* + MA9 or *Mx1-Cre;Celf2*^*fl/fl*^ + AML mice post-poly(I:C) injection (*n* = 8). **G** The percentage of GFP^+^ (left panel) or c-Kit^+^Gr-1^−^ (right panel) cells from BM in *Mx1-Cre* + MA9 or *Mx1-Cre;Celf2*^*fl/fl*^ + MA9 AML mice post-poly(I:C) injection (*n* = 4).

To determine how *Celf2* deletion accelerates leukemia initiation, we serially transplanted the equal numbers of *Celf2* KO + MA9 or MA9 leukemia cells into the recipient mice. The mice transplanted with *Celf2* KO + MA9 cells had more leukemic cells in the PB, BM and extramedullary organs than the controls three weeks after transplantation (Fig. S3A). Notably, over 40% of MA9 mice survived beyond 40 days after transplantation, while all *Celf2* KO + MA9 mice died within 22 days after transplantation (Fig. 3A), suggesting that *Celf2* deletion may increase the activity of LSCs.

The c-Kit^+^Gr-1^−^ leukemia cells were generally defined as LSCs in AML mouse model [18]. We found that the number and the percentage of c-Kit^+^Gr-1^−^ leukemia cells were increased in BM of *Celf2* KO + MA9 mice when compared with that of MA9 mice (Fig. 3B). Then, we performed the colony-forming assay and evaluated the self-renewal capacity of *Celf2* KO + MA9 LSCs in vitro. Three types of colonies were defined based on the literature: type A colonies had a compact center, type B colonies had a dense center surrounded by a halo of migrating cells, and type C colonies consisted of diffuse differentiating cells [19]. The results revealed that *Celf2* KO + MA9 leukemia cells gave rise to more colonies with larger size than MA9 leukemia cells, especially type A colonies (Fig. 3C, D), indicating that *Celf2* deletion increased the number of LSCs and colony-forming activity, as well as leukemic cell growth potential. We also found that the number of *Celf2* KO + MA9 cells was significantly increased than the number of MA9 cells (Fig. S3B). Consistent with more LSCs in the *Celf2* KO + MA9 cell population, extreme limiting dilution transplantation assays demonstrated a marked increase in leukemia-initiating cell frequency in *Celf2* KO + MA9 AML cells when compared with MA9 AML cells (Fig. 3E).

Next, we evaluated the consequence of *Celf2* loss in the maintenance of established AML leukemia. *Mx1-Cre* or *Mx1-Cre; Celf2*^*fl/fl*^ mouse cells were transformed by retrovirus containing MA9 and transplanted to lethally irradiated recipients. After 10 days, Cre expression and *Celf2* gene excision were induced by three intraperitoneal injections of poly(I:C) given every other day. The deletion of *Celf2* post-poly(I:C) induction was confirmed by PCR analysis and Western Blotting after BM aspiration (Fig. S3C-D). Deletion of *Celf2* significantly enhanced leukemia progression in MA9 mice (Fig. 3F). Consistent with the phenotypes of *Vav1-Cre; Celf2*^*fl/fl*^ + MA9 mice, the mice transplanted with *Mx1-Cre; Celf2*^*fl/fl*^ + MA9 cells had more leukemic cells in BM than the controls two weeks after transplantation (Fig. 3G, left panel). Among them, LSCs (c-Kit^+^Gr-1^−^) were also significantly increased (Fig. 3G, right panel). Taken as a whole, our results showed that *Celf2* loss enhanced the frequency and self-renewal capacity of MA9-transformed LSCs, accelerated the occurrence and the development of MA9-driven AML.

### *Celf2* deficiency activates leukemia related pathways

**Fig. 4:**
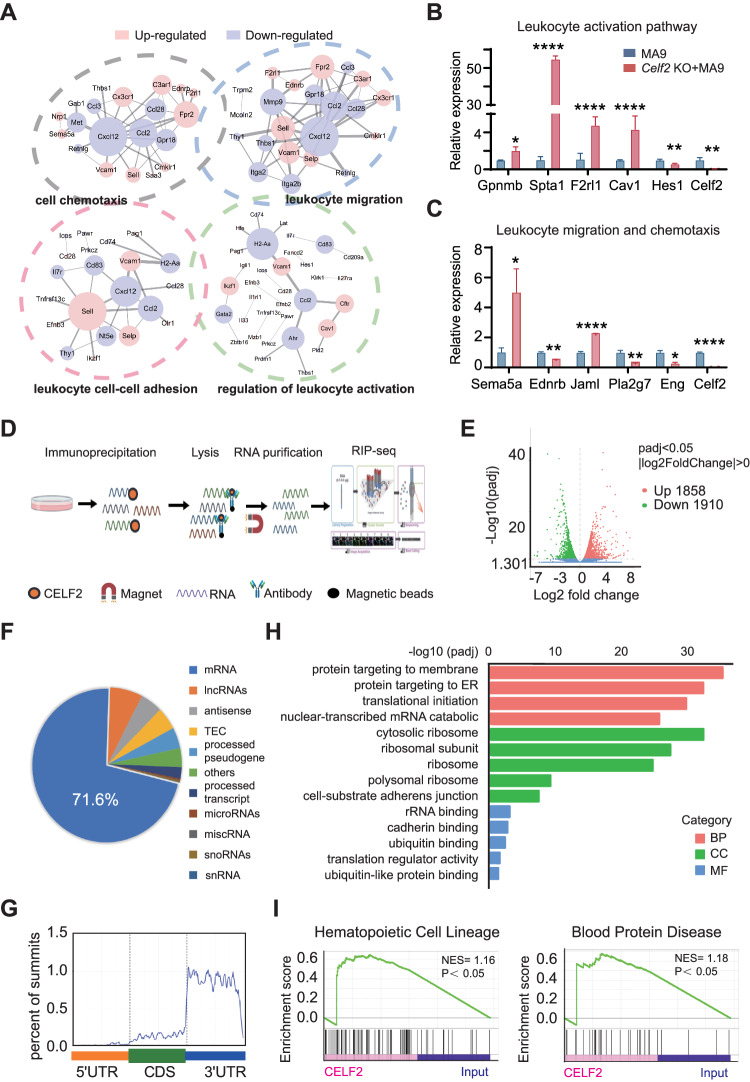
Transcriptional analysis of pathways in AML that are associated with *Celf2* deletion by RNA-Seq and RIP-Seq. **A** Gene ontology (GO) enrichment of four distinct signaling communities among RNA-Seq of MA9 or *Celf2* KO + AML mice. Levels of the pathway-related genes mRNA (**B**: leukocyte activation pathway; **C**: leukocyte migration and chemotaxis pathway) in MA9 or *Celf2* KO + AML leukemia cells were analyzed using RT-PCR. **D** Outline of the experimental methods for the CELF2 RIP-seq in leukemia cells. **E** Volcano plot of differentially enriched genes in RIP-Seq. The values of *X* and *Y* axes in the volcano plot are as the fold change (log_2_ transformed) and P adj (−log_10_ transformed) between two groups, respectively. Red/Green dots indicate 2-fold change differentially enriched genes with statistical significance. Blue dots indicate non-differentially enriched genes. **F** Percentages of various RNA species for RIP-seq of leukemia cells. **G** GO enrichment analysis of terms enriched in upregulated genes in RIP-Seq. **H** Metagene profiles of enrichment of CELF2-binding sites across mRNA transcriptome. CDS, coding sequence. **I** Enrichment plots from gene set enrichment analysis (GSEA) based on RIP-Seq data (left panel: hematopoietic cell lineage related genes; right panel: blood protein disease related genes) (normalized enrichment score [NES] is inferred from permutations of the gene set and the false discovery rate [FDR]).

To dissect the underlying molecular mechanism that drives the enhanced oncogenic potential in *Celf2* KO + MA9 cells, we performed global gene expression analysis of leukemia cells from *Celf2* KO + MA9 mice and the control MA9 mice by RNA-Seq. 1569 genes were found to be differentially expressed in leukemia cells with the presence or absence of *Celf2* (*p* < 0.05, |fold change| > 2) (Fig. S4). The differentially expressed genes could be classified mainly into four functional groups, including genes encoding products with cell chemotaxis, leukocyte migration, leukocyte cell-cell adhesion and leukocyte activation (Fig. 4A), suggesting that a variety of molecular and cellular processes have been altered in *Celf2* KO + MA9 cells. The representative top fold-changed genes were validated by Realtime-PCR, including those involved in leukocyte activation (*Gpnmb*, *Spta1*, *F2rl1*, *Cav1* and *Hes1*) and leukocyte migration (*Sema5a*, *Ednrb*, *Jaml*, *Pla2g7* and *Eng*) (Fig. 4B, C). Our data suggest that loss of *Celf2* likely enhances the self-renewal and survival of *Celf2* KO + MA9 cells through multiple molecular pathways that contribute to the MA9-induced leukemia.

To identify the molecular effectors that directly mediated by CELF2 in myeloid leukemia, we profiled the CELF2-binding RNAs at transcriptome level. We overexpressed 3xFlag-HA-CELF2 and performed RIP and high-throughput sequencing (RIP-seq) in K562 cells (Fig. 4D). Among the 1858 CELF2-dependent transcripts identified, 71.6% were mRNAs (Fig. 4E, F), and primarily were in the 3’ untranslated region (UTR) stem loop regions (Fig. 4G). GO analysis showed that CELF2-bound RNAs were enriched in various biological functions, such as membrane protein localization related to migration and adhesion, RNA degradation related to 3’UTR, translational initiation (Fig. 4H). GSEA analysis of these targets showed significant enrichment in pathways related to hematopoietic cell lineage and blood protein diseases (Fig. 4I), consistent with the essential role of CELF2 in hematopoiesis and leukemogenesis. Our RIP-seq results indicated that CELF2 may directly bind to multiple target mRNAs that participate in hematopoiesis and leukemogenesis

### Deletion of CELF2 enhances *FAT10* mRNA stability which activates the mTOR pathway, and promotes AML progression

**Fig. 5:**
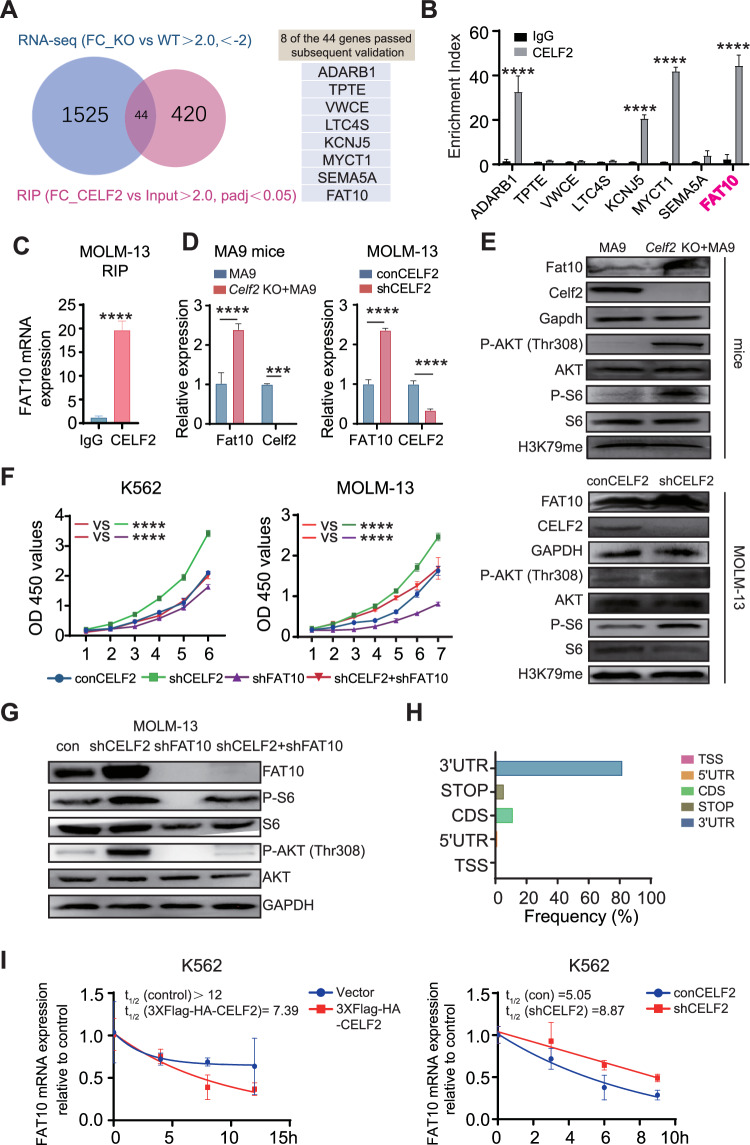
CELF2 mediates FAT10 mRNA degradation and inhibits AKT pathway activation. **A** Venn diagram illustrating the overlap of 44 mRNA targets of CELF2 identified using the RIP-seq and RNA-seq analysis (left panel); eight transcripts passed follow-up validation (right panel). **B** RT-PCR analysis of eight transcripts obtained from RIP with anti-CELF2 and IgG control antibodies in K562 cells. **C** RT-PCR analysis of FAT10 with anti-CELF2 and IgG control antibodies in MOLM-13 cells. **D** Levels of the *Fat10* (*FAT10*) mRNA in MA9 mice (left panel) and MOLM-13 (right panel) were analyzed using RT-PCR. **E** Levels of pathway-related proteins in Celf2 (CELF2) knockdown leukemia cells (upper panels: mice; lower panels: MOLM-13) were analyzed by Western blot. **F** Cell proliferation in the indicated cell lines after transduction with lentivirus expressing control, *CELF2*-specific shRNA, *FAT10*-specific shRNA or *CELF2*-specific shRNA and *FAT10*-specific shRNA. **G** Levels of pathway-related proteins in MOLM-13 after transduction with lentivirus expressing control, *CELF2*-specific shRNA, *FAT10*-specific shRNA or *CELF2*-specific shRNA and *FAT10*-specific shRNA. **H** The distribution of CELF2-binding peaks within different gene regions identified by FLAG-CELF2 RIP-seq in K562 cells. **I** After treatment with 10 mg/mL Actinomycin-D, total RNA was extracted at specific time. The half-lives of *FAT10* mRNA in CELF2-overexpressing K562 cells and CELF2 knockdown K562 cells were measured. Panels **B**–**F**, **H** show 1 representative of at least 3 independent experiments.

To specifically define the downstream signals of CELF2 that are critical for leukemia progression, we analyzed integrated data from RIP-Seq and RNA-Seq to identify the target mRNA of CELF2. Using the criteria of padj < 0.05 and fold-change value > 2, we identified 464 mRNA targets of the CELF2 protein in RIP-Seq and 647 genes that were differentially expressed above the threshold level in RNA-seq of leukemia cells from *Celf2* KO + MA9 mice and the control MA9 mice. 44 putative mRNA targets were obtained via the integrated data (Fig. 5A). Of these, using qRT-PCR, 8 genes were found to be both enriched in RIP and dysregulated by CELF2 in AML cells (Fig. 5B). *FAT10* (Human Leukocyte Antigen-F adjacent transcript 10) mRNA was found to be highly enriched by CELF2 RIP, and its mRNA and protein were significantly upregulated in *Celf2* KO AML mice and cells (Fig. 5B–E). We observed that *FAT10* was highly expressed in AML patient samples when compared with normal individuals (Fig. S5A). Since FAT10 has been reported to directly promote cancer cell proliferation in various cancers by activating the AKT-mTORC1 signaling pathway [20], we examined the levels of FAT10, P-AKT (Thr308), P-S6 and H3K79me in *Celf2* KO + MA9 AML mouse cells and *CELF2* KD human MOLM-13 and K562 myeloid leukemia cells. We found that the levels of FAT10, P-AKT (Thr308) and P-S6 were significantly increased in both murine and human cells when compared with *Celf2* WT + MA9 AML cells and *CELF2* WT human MOLM-13 and K562 myeloid leukemia cells respectively, while the level of H3K79me was equivalent in *Celf2 KO* + MA9 and MA9 AML cells (Figs. 5E and S5B). Interestingly, the level of P-AKT in *Celf2* KO HSCs and control HSCs measured by flow cytometry did not show significantly increased in *Celf2* KO HSCs when compared with that of controls (Fig. S5C). We further knocked down FAT10 in MOLM-13 and K562 cells, observed that FAT10 KD reduced the P-AKT (Thr308) and P-S6 level (Fig. S5D). Our results thus demonstrated that FAT10 expression reduction could inhibits AKT pathway activation in AML.

To further investigate whether CELF2 regulates AKT-mTORC1 signaling pathway via FAT10, FAT10 was knocked down in CELF2 KD MOLM-13 and CELF2 KD K562 cells. We found that decreased FAT10 expression inhibited the over-growth of CELF2 KD myeloid leukemia cells, reversed the effect of *CELF2* deletion (Fig. 5F). Notably, the phosphorylation levels of P-AKT (Thr308) and P-S6 were significantly reduced in FAT10 KD MOLM-13 and FAT10 KD K562 cells (Figs. 5G and S5E). We further knocked down FAT10 (FAT10 KD) in CELF2 KD MOLM-13 cells and transplanted the cells to BALB/c nude mice to determine the cell proliferation in vivo. We found that FAT10 KD could inhibit the growth of CELF2 KD MOLM-13 cells when compared with the control group (Fig. S5F).

We further analyzed the relationship between CELF2 and FAT10 in AML patients and normal individuals from dataset (LAML tumor and LAML normal) in GEPIA (Gene Expression Profiling Interactive Analysis) (http://gepia.cancer-pku.cn/detail.php?gene=CELF2). We found that the expression of CELF2 is negatively correlated with the expression FAT10. The expression of FAT10 is high in BM cells with CELF2 low expression (Fig. S5G). Taken together, these results demonstrated that loss of CELF2 accelerated the myeloid leukemia cell proliferation by activating the FAT10-AKT-mTORC1 signaling pathway.

To investigate how CELF2 regulates FAT10, we examined the location of the mRNA compete-derived consensus motif and found that CELF2 was highly enriched in 3′UTRs of targeting mRNA (Fig. 5H). We further analyzed the raw data of RIP-seq and the IGV peaks for the binding sites of CELF2 on FAT10 (Fig. S5H). Our results showed that CELF2 is usually enriched in the FAT10 3′UTR region. Additionally, we found that over-expression of CELF2 significantly promoted the degradation of *FAT10* mRNA in K562 cells following actinomycin-D (10 μg/mL) treatment, while the *FAT10* transcripts exhibited longer half-lives in CELF2 KD K562 cells (Fig. 5I). These results suggested that CELF2 directly fine-tunes the transcription of FAT10, thus inhibits the FAT10-AKT-mTORC1 signaling pathway in myeloid leukemia cells.

In addition, The AU-rich elements (AREs) in 3’UTRs are the most prevalent cis-destabilizing RNA motifs [21, 22]. ARE-containing mRNA transcripts are considered to be inherently unstable, and are subjected to dynamic decay for mRNA circulation [21]. Previous studies indicated that CELF2 could regulate target gene expression by binding to AREs in the 3′UTR of its target mRNAs [23–25]. By analyzing the sequence of FAT10 3’UTR region, we found three AREs (Fig. S6A). We constructed the luciferase (Fluc) reporter that was inserted with different AREs of FAT10 and examined the Fluc activity in CELF2 KD K562 cells and controls (Fig. S6B). We found that CELF2 KD induced a significantly increase in Fluc activity of FAT10 reporter (Fig. S6C), indicating that CELF2 are likely binding to the last AREs in 3′UTR of FAT10. In summary, we identified the 3′UTR of FAT10 mRNA contains ARE regions as a potential functional CELF2-binding site, which may contribute to the decay of FAT10 mRNA.

### Combined treatment with EPZ-5676 and Rapamycin shows synergistic anti-tumor effects in *Celf2* KO + MA9 mouse model

**Fig. 6:**
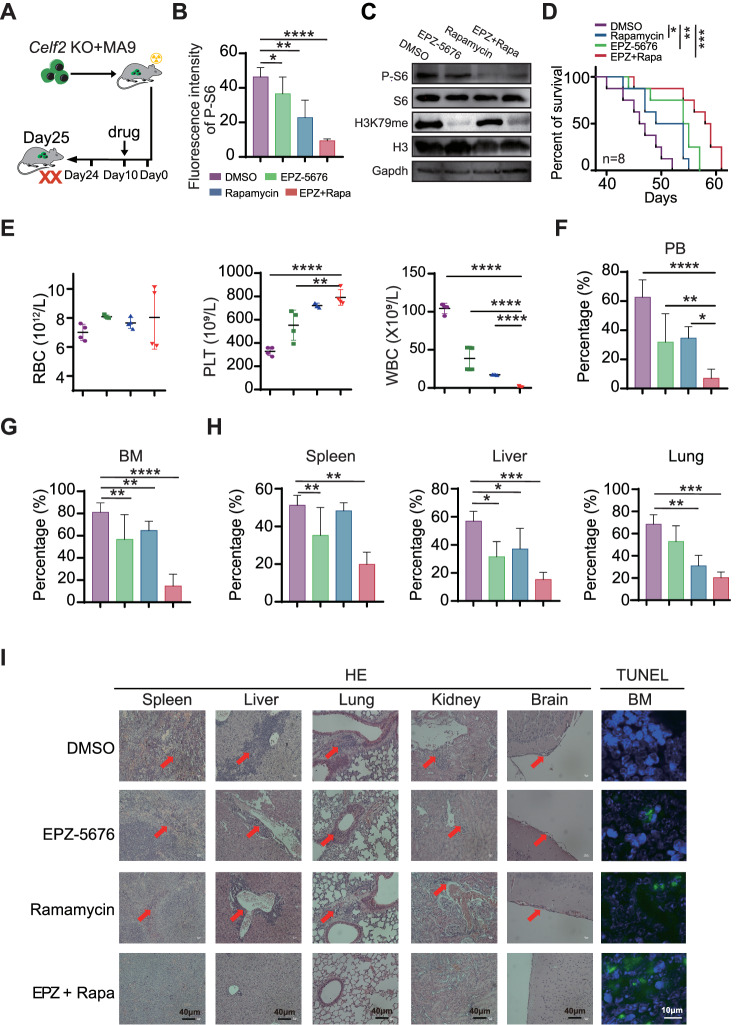
Combined treatment with EPZ-5676 and rapamycin prolonged the survival of *Celf2* KO + MA9 mice. **A** Schematic representation of different drug treatments in *Celf2* KO + MA9 mice. **B** Phospho-flow cytometry was used to measure the phosphorylation level of S6 in leukemia cells from *Celf2* KO + MA9 mice treated with placebo, single EPZ-5676, single rapamycin, or combined EPZ-5676 and rapamycin through tail vein injection (*n* = 5). **C** Western blotting was used to assess the expression of P-S6, S6, H3K79me, H3K79 in leukemia cells from *Celf2* KO + MA9 mice treated with different drugs. **D** Kaplan–Meier survival curves of *Celf2* KO + MA9 mice treated with different drugs (*n* = 8). **E** The counts of RBC, PLT and WBC in PB by routine blood tests. The percentage of GFP^+^ leukemia cells in PB (**F**) and BM (**G**) from *Celf2* KO + MA9 mice treated with different drugs (*n* = 5). **H** The percentage of GFP^+^ leukemia cells in organs (spleen, liver, lung) from *Celf2* KO + MA9 mice treated with different drugs (*n* = 5). **I** Histological staining for hematoxylin and eosin (HE) in spleen, liver, lung, kidney and brain (magnification, ×40), and TUNEL staining in BM (green represents TUNEL, blue represents DAPI, magnification, ×60), from *Celf2* KO + MA9 mice treated with different drugs.

Since FAT10-mTORC1 signaling pathway plays a central role in the pathogenesis of *Celf2* KO + MA9 AML, we sought to determine whether dual inhibition of mTORC1 and MA9 pathways would be beneficial for *Celf2* KO + MA9 AML mice. Rapamycin is a mTOR inhibitor while pinometostat (EPZ-5676) is a potent DOT1L histone methyltransferase inhibitor which could effectively inhibit MLL-fusion target gene expression in vivo. *Celf2* KO + MA9 mice were treated with either placebo, single EPZ-5676, single rapamycin, or combined EPZ-5676 and rapamycin (Fig. 6A). We found that the levels of P-S6 and H3K79me in *Celf2* KO + MA9 cells were significantly decreased, while the protein levels of S6 and H3 were equivalent in BM cells (Figs. 6B, C and S7A). The survival time of the mice treated with EPZ-5676+rapamycin was significantly longer than that of mice treated with other treatment (Fig. 6D). The counts of PLT were increased, while the count of WBCs was decreased in the PB of mice treated with EPZ-5676+rapamycin when compared with that of the mice treated with single EPZ-5676 or rapamycin only (Fig. 6E). Additionally, the percentages of GFP^+^ leukemia cells in the PB, BM, spleen, liver and lung were decreased in mice treated with EPZ-5676+rapamycin when compared with those in other groups (Fig. 6F, H). HE staining showed that the degree of leukemia cell infiltration in the spleen, liver, lung, kidney and brain was reduced in mice treated with EPZ-5676+rapamycin when compared with other groups (Fig. 6I, HE panels). Also, immunohistochemical staining of Ki67, a marker of cell proliferation, showed significantly reduced staining in the EPZ-5676+rapamycin-treated group when compared with the other groups (Fig. S7B), while TUNEL (TdT-mediated dUTP nick-end labeling) staining, an indicator of apoptotic cells, was shown to be increased in mice treated with EPZ-5676+rapamycin when compared with the other groups (Fig. 6I, TUNEL panels). Thus, our results showed that combined therapy with EPZ-5676 and rapamycin could reduce *Celf2* KO + MA9 leukemia burden and prolong the survival time of *Celf2* KO + MA9 AML mice by inhibiting both mTORC1 pathway and MLL-fusion target gene expression.

## Discussion

Dysregulation of RBPs was common in some of the aggressive myeloid leukemia AMLs that are characterized by abnormal growth and differentiation of hematopoietic stem cells (HSCs). The alterations in RBPs establish a dysregulated epigenetic state which is a key event in leukemia pathogenesis within myeloid progenitors [26, 27]. CELF2 is proposed to participate in post-transcriptional modification to regulate the stability or alternative splicing of target mRNA [15, 28]. In this study, we delineated the role of CELF2 in hematopoiesis and leukemogenesis.

The development of AML is a stepwise process via the accumulation of somatic mutations or alterations. Although numerous studies have identified t(11q23)/MLL as a driver mutation that converts HPCs into pre-leukemic stem cells in AML initiation [29–34], the patients with t(11q23)/MLL usually presented with multiple gene alterations at diagnosis, suggesting that other associated mutations might cooperate with t(11q23)/MLL in driving/assisting AML progression [31, 35, 36]. In the current study, using hematopoietic-specific *Celf2* deletion murine AML models, we observed a strong cooperation between inactivation of Celf2 and MLL-AF9 leading to rapid MA9-induced myeloid leukemia initiation and exacerbated AML progression. Interestingly, although our data demonstrated that *Celf2* deficiency slightly enhanced HSCs self-renewal and differentiation to myeloid cells in vivo, the deficiency alone could not cause leukemia in the aged mice (data not shown), suggesting that CELF2 may function as a cell growth balancer under normal condition in vivo.

CELF2 has been shown to regulate the mRNA translation and stability by binding to sequence motifs in the 3′UTR in several studies. For example, CELF2 repressed *TBR2* mRNA translation via the 3′UTR and instructed neural stem cell fates [37]. In cancerous human epithelial cells, CELF2 could bind to the *COX-2* 3′UTR and inhibited its translation [38]. These studies suggested that CELF2 may exert its functional role by regulating the stability of target RNA. In this study, we demonstrated that CELF2 directly bound to the *FAT10* mRNA and regulated its stability.

Interestingly, we found that the level of H3K79me, the key downstream symbol of classical pathway of MLL-AF9, was equivalent in *Celf2* KO + MA9 and MA9 AML cells (Figs. 5E and S5B) [39–42], while the mTORC1 signaling pathway was activated only in *Celf2* KO + MA9 but not in *Celf2* WT + MA9 leukemia cells (Fig. 5). This suggested that the activation of FAT10-mTORC1 signaling pathway caused by loss of *Celf2* is independent to the MA9-DOT1L signaling pathway. FAT10 belongs to the ubiquitin-like protein family and was demonstrated to be able to increase AKT phosphorylation, and thereby activate the mTORC1 signaling pathway [43–46]. In Yan’s study, FAT10 was shown to activate AKT signaling by stabilizing EGFR in bladder cancer cells [44]. In Rongfa’s study, they revealed that FAT10 directly interacted with β-catenin and inhibited its ubiquitin-dependent degradation [47]. Although AKT has been identified as a critical downstream target of FAT10, the exact molecular mechanism(s) by which FAT10 specifically regulated AKT needs additional independent studies to delineate.

Consistent with previous studies showed that hyper-activation of FAT10 increased the potential of tumorigenesis [20, 48, 49], we demonstrated that loss of *Celf2* increased MA9-induced AML initiation by upregulating FAT10 expression, thus increasing AKT phosphorylation, and subsequent activation of mTORC1 (Fig. 5). Indeed, we found that *Celf2* KO + MA9 AML cells were more sensitive to combined treatment of Rapamycin [50, 51] and EPZ-5676 [52, 53]. It will be interesting to assess whether AML patients with t(11q23)/MLL and *CELF2* deficiency could further benefit from combined therapy with the MA9/DOTL1 inhibitor and the mTORC1 inhibitor.

**Fig. 7:**
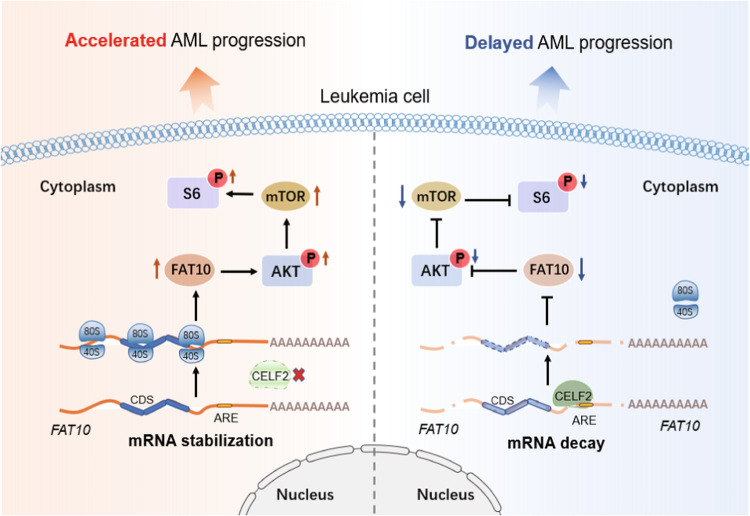
Schematic depicting the tumor suppressive role of CELF2 in MA9-induced AML. In normal hematopoiesis, CELF2 binds to FAT10 mRNA, promotes the decay of mRNA, and suppresses the mTORC1 signaling pathway. In MLL-AF9-induced AML, CELF2 deletion increases the stability of FAT10 mRNA, promotes the phosphorylation of AKT, activates mTORC1 pathway, and exacerbates the progression of MLL-AF9 induced AML.

In summary, we found that CELF2 regulates AML initiation and progression by post-transcriptional regulation (Fig. 7). Loss of CELF2 inhibits mRNA decay of FAT10 and activates AKT/mTORC1 signaling pathway in AML, independent of the MA9/DOTL1 signaling pathway. CELF2-FAT10-mTOR axis is a potential therapeutic target in myeloid leukemia suppression.

## Materials and methods

### Generation of the *Celf2*-deficient murine MLL-AF9 leukemia model

*Celf2* conditional knockout mice were generated by crossing *Celf2*-floxed mice with *Mx1-Cre* or *Vav1-Cre* transgenic mice. BM cells were isolated from 8- to 12-week-old *Celf2* conditional knockout mice and corresponding control mice, and lineage-negative cells (Lin^–^) were enriched from bone marrow cells and were used for generating MLL-AF9 AML mice. Experimental details are provided in the supplemental data.

### MLL-AF9 murine leukemia treatment study

Lin^−^ cells were isolated from 8-week-old *Vav1-Cre;Celf2*^*fl/fl*^ and *Celf2*^*fl/fl*^ mice, transduced with supernatant containing retroviruses encoding MLL-AF9 and GFP to generate MLL-AF9 AML mice. On day 10 post transplantation, the mice were randomly split into 4 groups and mice in each group were treated with 10 mg/kg EPZ-5676, 5 mg/kg Rapamycin, 10 mg/kg EPZ-5676 + 5 mg/kg Rapamycin or control for 20 days respectively, once every 2 days. On day 24, all mice were sacrificed and AML burden was assessed by FACS in peripheral blood, spleen and BM. Moribund mice were euthanized and analyzed for their leukemia burden as well.

### RNA immunoprecipitation

K562 cells with either expression of 3xflag-HA-CELF2 or empty vector were seeded in 10 cm dishes, and harvested. Five micrograms of CELF2 antibody or rabbit IgG was conjugated to protein A/G magnetic beads by incubation for 4 h at 4 °C, and then washed three times with wash buffer (20 mM HEPES pH 7.9, 150 mM NaCl, 10 mM KCl, 1.5 mM MgCl_2_, and 0.5 mM EDTA pH 8.0, 0.5% NP-40, 10% Glycerol, 1.5 mM DTT, and protease inhibitor) and incubated with pre-cleared nuclear extraction in lysis buffer (wash buffer + 10 U/ml RNase inhibitor) at 4 °C overnight. After washing with wash buffer for three times, beads were resuspended in 50 μl of PBS, followed by DNA digestion at 37 °C for 15 min. Supernatants were taken and western blotting analysis were performed respectively. Input and co-immunoprecipitated RNAs were recovered by TRIzol, and extraction was analyzed by qPCR or RNA-seq.

### Statistical analysis

Kaplan–Meier survival curve *p* values were performed using Log-rank Mantel–Cox test. For statistical comparison, unpaired Student’s *t* test was used. Statistical analyses were performed using Prism 7 software (GraphPad). Data with statistical significance are as indicated, *p < 0.05, **p < 0.01, ***p < 0.001.

### Supplementary information

Supplementary File

Supplementary Table 1

Supplementary Table 2

## Data Availability

For original data, please contact wpyuan@ihcams.ac.cn. The accession numbers for the RNA-seq and RIP-seq data reported in this research were deposited in GEO under the accession number GEO: GSE217989.
