# Supplementary material for: Loss of RNA-binding protein CELF2 promotes acute leukemia development via FAT10-mTORC1
Source: Oncogene. 2024 Mar 21;43(19):1476–87. doi: 10.1038/s41388-024-03006-3 (PMC11068570; doi:10.1038/s41388-024-03006-3)
Supplement: Supplementary file 1 — Supplementary File [file 41388_2024_3006_MOESM1_ESM.docx]

**Supplemental data file for:** **Loss of RNA binding protein CELF2 promotes acute leukemia development via FAT10-mTORC1**

Tengxiao Guo^1,2^, Yuxia Wang^1^, Xiaolu Sun^1^, Shuaibing Hou^1^, Yanjie Lan^1^, Shengnan Yuan^1^, Shuang Yang^1,2^, Fei Zhao^1,2^, Yajing Chu^1,2^, Yuanwu Ma^3^, Tao Cheng^1,2^, Jia Yu^4^, Bing Liu^5^, Weiping Yuan^1,2*^ and Xiaomin Wang^6,7*^

Supplemental Materials and Methods

Analysis of TCGA datasets

Data mining from the publicly available TCGA acute myelocytic leukemia samples report was performed for AML patients. All genomic data analyzed herein were generated by the TCGA Research Network (http://www.cbioportal.org/). We analyzed the data for CELF2 expression from 200 AML patient samples.

Primary Human Samples

Bone marrow (BM) samples of 9 newly diagnosed AML patients and 7 normal donors were collected from Blood Diseases Hospital, Chinese Academy of Medical Sciences and Peking Union Medical College (CAMS & PUMC) with informed consent (ethical review committee approval number KT2020011-EC-2).

Mice generation and genotyping

*Celf2* conditional deletion mice were generated using the homologous recombination technique to insert two loxp sites franking the exon 5 of *Celf2* gene. (Supplemental Figure 1A). The mice were then mated with *Vav1-Cre* transgenic mice expressing Cre recombinase under the control of the *Vav1* promoter. This will cause a deletion of exon 5 that results in a stop codon to delete *Celf2* in hematopoietic cells at the embryonic stage. The breeding scheme of *Mx-1* conditional knockout mice is the same as above. Mice in which *Celf2* was deleted in the hematopoietic system (*Vav1-Cre;Celf2^fl/fl^* or *Mx-1-Cre;Celf2^fl/fl^*), the *Celf2^fl/fl^* mice and the control C57BL/6 mice (6-8 weeks old) were maintained at the specific pathogen-free (SPF) animal facility of the State Key Laboratory of Experimental Hematology (SKLEH). All animal experiments and surgeries were approved by the Institutional Animal Care and Use Committee (IACUC), Institute of Hematology and Blood Diseases Hospital, CAMS/PUMC. All efforts were made to minimize mouse suffering.

Flow cytometry analysis

A 20 μl peripheral blood (PB) sample was obtained from either tail vein or retro-orbital bleeding and were diluted with PBE (PBS containing 2% fetal bovine serum and 20 mM EDTA). Before staining, red blood cells (RBCs) were lysed via ammonium chloride-potassium bicarbonate. Bone marrow (BM) cells were flushed from tibias, femur and ilium with PBE. The spleen, liver and lung were ground and passed through the yellow membrane, and the cells were collected. The cells were stained with the following antibodies: anti-mouse CD3 PE-Cy7, anti-mouse CD4 PE-Cy7, anti-mouse CD8 APC-Cy7, anti-mouse Mac-1 APC-Cy7, anti-mouse B220 APC, anti-mouse c-Kit APC, anti-mouse Gr-1 PE, anti-mouse Mac-1 APC-Cy7, anti-mouse GFP FITC, anti-mouse CD150 PE, anti-mouse CD3 biotin, anti-mouse CD4 biotin, anti-mouse CD8 biotin, anti-mouse TER-119 biotin, anti-human/mouse CD45R (B220) biotin, anti-mouse CD127 PE, anti-mouse Sca-1 PE-Cy7, anti-mouse CD34 FITC, anti-mouse FLK2(CD135) PE, anti-mouse CD48 FITC, anti-mouse CD16/32 PE. Flow cytometry was performed on LSR Ⅱ (BD Biosciences) and live cells were gated based on FSCA and SSC-A characteristics. Data were analyzed with FlowJo software. Flow cytometric sorting was conducted using a FACS Aria (BD Biosciences).

Transplantation and chimerism assessment

Competitive transplantations were performed with 1 million BM cells from 8-week-old *Vav1-Cre;Celf2^fl/fl^* or *Celf2^fl/fl^* mice and 1 million BM cells from 8-week-old B6SJL mice, injected into lethally irradiated B6SJL congenic CD45.1 recipients. In the primary transplants, chimerism was checked from 4 to 16 weeks, every 4 weeks by either assessing chimerism via peripheral blood or bone marrow aspirates. Mice were sacrificed 16 weeks after injection for primary transplant and secondary transplant experiments.

Generation of the murine MLL-AF9 leukemia model

BM cells were extracted from 8-week-old *Celf2* conditional knockout and wild-type (WT) mice, and lineage-negative cells (Lin^–^) were enriched and used for generating MLL-AF9 AML mice. For primary engraftment of transformed MLL-AF9 leukemic cells in leukemia initiation experiments, 1,000,000 leukemic cells were injected into 8-week-old C57BL/6 female mice, which were lethally irradiated with 9 Gray. In secondary and tertiary transplants, cell numbers of 500,000 and 100,000 bone marrow leukemic cells were transplanted into C57BL/6 mice sublethally irradiated with 4.5 Gray respectively. MLL-AF9 leukemia maintenance experiments were performed by injecting MLL-AF9 WT and *Celf2* KO+MA9 cells into sublethally irradiated mice, and treating the mice with 10 mg/kg EPZ-5676, 5 mg/kg rapamycin or placebo dosing once every two days respectively.

Approximately 5 × 10^6^ MOLM-13 cells (con, shCELF2, shFAT10, shCELF2 + shFAT10) per mouse suspended in 100 μl PBS were injected in the flank of male BALB/c nude mice (6 weeks old). During the 30-day observation, the tumor size (V = (width^2^ × length × 0.52)) was measured with vernier caliper.

Cell culture

K562 (human chronic myeloid/erythron leukemia cell line), MOLM-13 (human acute myeloid leukemia) and THP-1 (acute monocytic leukemia) leukemia cells were cultured in RPMI/10% fetal calf serum (FCS, heat inactivated). All cell lines were grown in the recommended cell culture media at 37℃ in 5% CO_2_. None of the cell lines above were listed in the database of commonly misidentified cell lines maintained by ICLAC and NCBI BioSample. Cell lines were authenticated using short-tandem repeat (STR) assays at the Characterized Cell Line Facility at MD Anderson Cancer Center. For cell apoptosis assay in MOLM-13 and THP1, cells were starved in serum-free medium for 48 hours before Flow cytometry analysis.

Plasmid construction

*CELF2* was PCR amplified from human cDNA and cloned into MSCV Puro-IRES-GFP construct. 3xflag-HA-CELF2 retroviral overexpression plasmid was purchased from JIKAI. K562 cells were transduced with either empty control vector or 3xflag-HA-CELF2 construct, and high GFP^+^ expressing cells were sorted for *in vitro* experiments.

CELF2- or FAT10-knockdown cell lines were established. The CELF2 shRNA sequences, C1 (5′-GAATGCACTGCACAATATT-3′, accession number NM_001025076), C2 (5′-CACCTATCGTGGTGAAGTT-3′, accession number NM_001025076), and C3 (5′- CACAGTATCTGGCGCTCCT-3′, accession number NM_001025076) were cloned into the pLKO.1 backbone at the AgeI and EcoRI restriction sites(1). The sequences of the shRNA target for FAT10 mRNA are 5′- GGCAGATTACGGCATCAGA -3′, 5′- GGAGAAGCCTCTCATCTTA -3′. All siRNAs were purchased from Sigma-Aldrich.

Quantitative real-time PCR.

RNA was extracted using the RNeasy Mini Kit (QIAGEN, 74106, Germany) according to the manufacturer’s protocol. cDNA synthesis was performed using a cDNA reverse transcription kit (Takara, RR047A, Japan) according to the manufacturer’s protocol. Quantitative PCR assays were performed in 96-well Micro Amp Fast Optical 96-Well Reaction Plates (Applied Biosystems, 4344904, USA) using SYBR Green Mix (Roche, 04913914001, Switzerland). The signal was detected using the Step-One Plus Real-Time PCR System. GAPDH was used as an endogenous control for gene expression assays. Primers used in reverse-transcriptase polymerase chain reactions (RT–PCR) were: M-CELF2-ko-exon5-forward: TTGAGGCCCAGAATGCACTG, M-CELF2-ko-exon5-reverse: AACATCACTCTGATATCATTCTCGT; Forward-HUMAN-CELF2: TGACCTCTCTCGGGACTCTG, Reverse-HUMAN-CELF2: ATTCAGAGCCGCCATACCTG.

Cell sorting

For mice BM LSK cell sorting, BM cells were enriched for Lin^-^ cells using Lin^-^ microbeads (130-090-858; Miltenyi Biotec) and an Auto MACS cell separator (Miltenyi Biotec), and were subsequently stained with anti-mouse c-Kit APC and Ly-6A/E(Sca-1) PE-Cyanine7 to sort LSKs. Cell isolation was performed on a FACS Aria-II (Becton Dickenson).

RNA stability assays

Stability of mRNA was assessed following the addition of actinomycin D (10 µg/mL), a potent inhibitor of mRNA synthesis. After 0 to 12 hours of actinomycin D treatment, total mRNA was extracted and subjected to quantitative polymerase chain reaction (qPCR). Data are presented relative to control cells at the time of addition of actinomycin D.

Western blotting analysis

Western blotting analyses were carried out as previously described (Melton et al., 2007). Briefly, 10 mg of total protein lysates were loaded into 10% 37.5:1 bis-acrylamide SDS-PAGE gels. Antibodies used for Western blots were as follows: CELF2 (Abcam, ab186430), ANTI-FLAG® M2 Affinity Gel (Sigma, A2220-1ML), Dynabeads™ Protein A/G for Immunoprecipitation (Invitrogen™, 10001D).

Statistical analysis

Kaplan-Meier survival curve p values were performed using Log rank Mantel-COX test. For statistical comparison, unpaired Student’s t test was used. Statistical analyses were performed using Prism 7 software (GraphPad). Data with statistical significance are as indicated, *p< 0.05, **p< 0.01, ***p< 0.001.

Data availability

The accession numbers for the RNA-seq and RIP-seq data reported in this paper were deposited in GEO under the accession number GEO: GSE217989.

Supplemental Figure 1


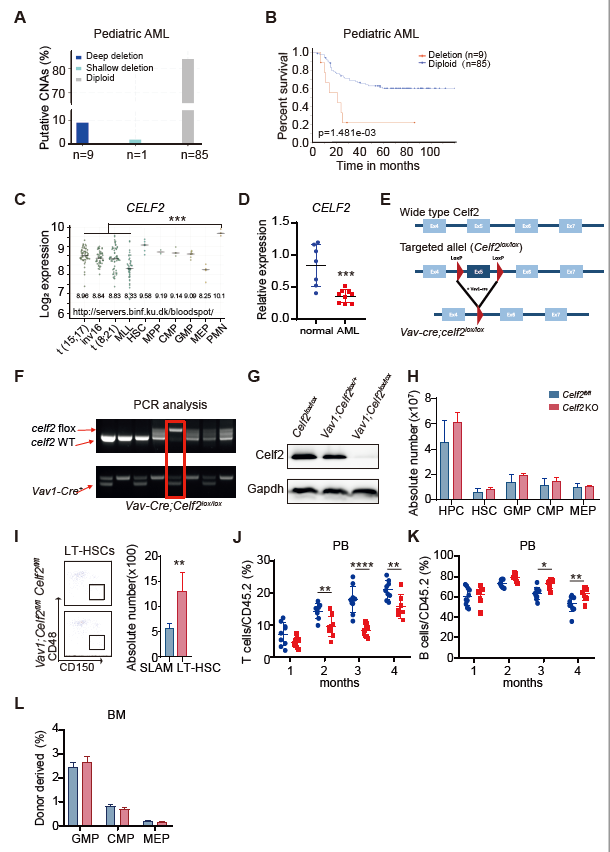


Supplemental Figure 1. CELF2 may play an important role in normal hematopoiesis and leukemogenesis in AML patients.

(A) Histogram of putative copy-number alternation in Pediatric Acute Myeloid Leukemia of CELF2 based on the TCGA. (B) Kaplan–Meier plot for Pediatric Acute Myeloid Leukemia patients with copy number deep deletion of the CELF2 gene compared to the diploid. (C) The mRNA expression of CELF2 in AML cells of different karyotypes and normal hematopoietic cells (Data source: GSE42519 for normal hematopoietic cells and GSE13159 for AML cells). (D) Expression of the *CELF2* mRNA in 9 AML patient samples and 7 normal individuals analyzed using RT-PCR. (E) Scheme for generating *Celf2* hematopoietic system conditional knockout mouse. (F) PCR analysis showing the efficiency of *Celf2* deletion. (G) Western blot analysis showing Celf2 levels in BM. (H) The percentage of donor-derived GMPs, CMPs and MEPs compartments in BM of recipients at 16 weeks after competitive BMT (n=7). (I) The total number of LT-HSCs identified by SLAM markers. (J-K) Flow cytometry analysis of the percentages of *Celf2* WT and *Celf2* KO donor-derived T and B cells in the PB of recipient mice after competitive BMT (n=7). (L) The percentage of donor-derived GMPs, CMPs and MEPs compartments in BM of recipients at 16 weeks after competitive BMT (n=7). The p-values were determined using a log-rank Mantel-Cox test (****p < 0.0001).

Supplemental Figure 2


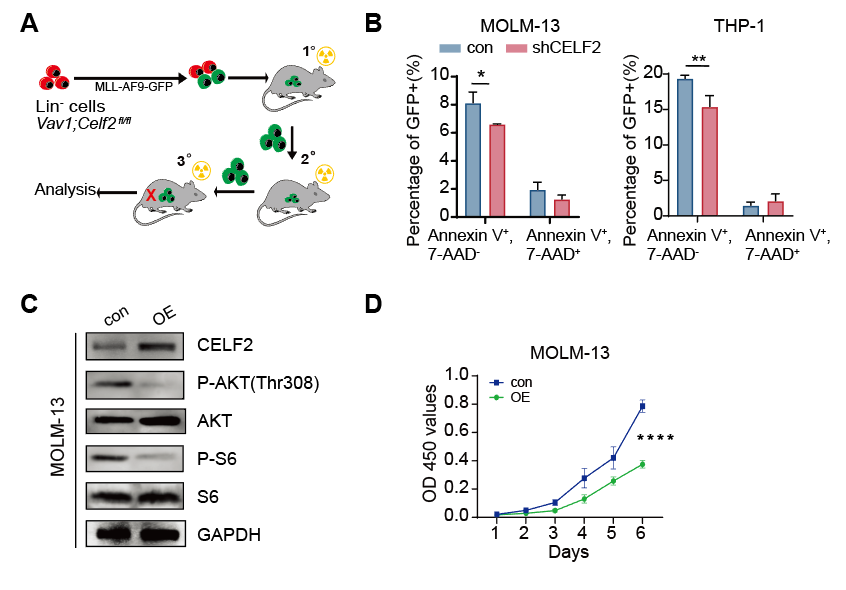


Supplemental Figure 2. CELF2 deficiency inhibits apoptosis of leukemia cells.

(A) Schematic of MLL-AF9 retroviral BM transplantation model. Lin^-^ BM cells were transduced with MLL-AF9-GFP retrovirus, and transplanted into sublethally irradiated recipient mice. GFP^+^ leukemia cells from AML mice were sorted and used for experiments as indicated. (B) Percentages of apoptotic cells in MOLM-13 and THP-1 cell lines. (C) The expression of CELF2 and P-AKT in CELF2 OE MOLM-13 (OE: overexpression). (D) The proliferation of CELF2 OE and control MOLM13 cells.

Supplemental Figure 3


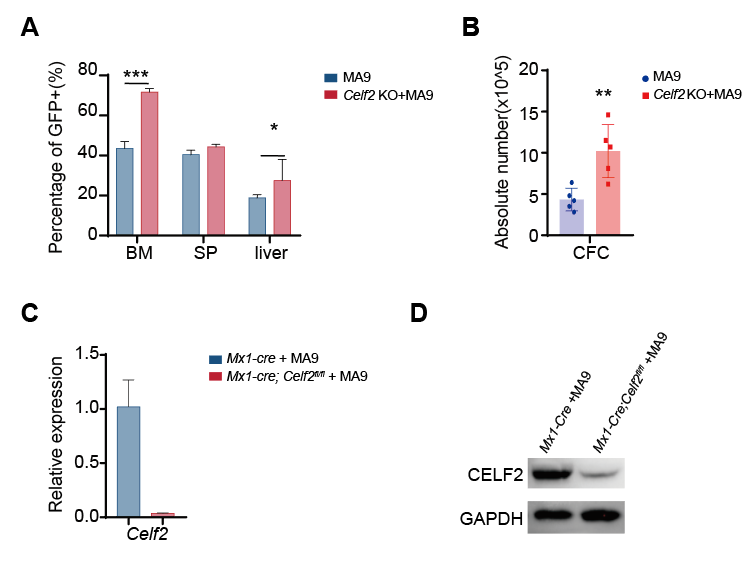


Supplemental Figure 3. CELF2 deficiency accelerated the occurrence and the development of MA9-driven AML.

(A) The percentage of GFP^+^ cells in bone marrow, spleen and liver of MA9 or *Celf2* KO+MA9 mice in tertiary transplant (n=4). (B) The absolute number of cells in colonies. (C) Expression of the *Celf2* mRNA in *Mx1-Cre* +MA9 or *Mx1-Cre;Celf2^fl/fl^* +MA9 mice BM cells post-poly(I:C) induction were analyzed using RT-PCR. (D) The protein level of CELF2 in BM cells from *Mx1-Cre* or *Mx1-Cre;Celf2^fl/fl^* mice post-poly (I:C) induction.

Supplemental Figure 4


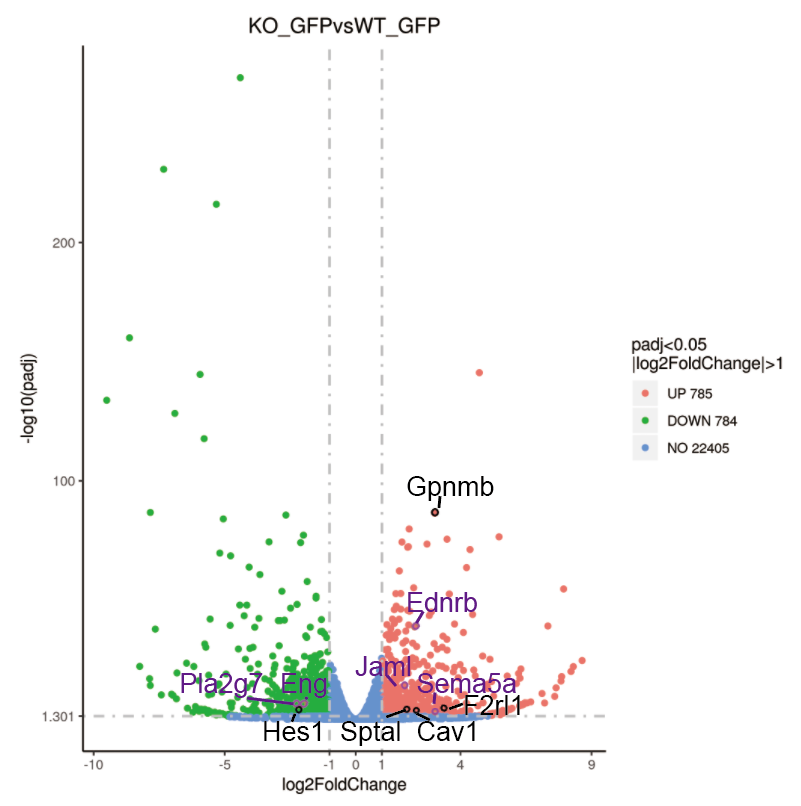


Supplemental Figure 4. Transcriptional analysis of RNA-Seq

Volcano plot of differentially expressed genes in RNA-Seq. The values of X and Y axes are the fold change (log_2_ transformed) and P adj (−log_10_ transformed) between the leukemia cells from Celf2 KO+MA9 mice and control MA9 mice respectively. Red/Green dots indicate 2-fold change differentially expressed genes with statistical significance. Blue dots indicate non-differentially expressed gene. The RT-qPCR-validated genes shown in Fig.4B and 4C were also indicated in this figure. Black circles indicate leukocyte activation pathway related genes. Purple circles indicate leukocyte migration and chemotaxis pathway related genes.

Supplemental Figure 5

**
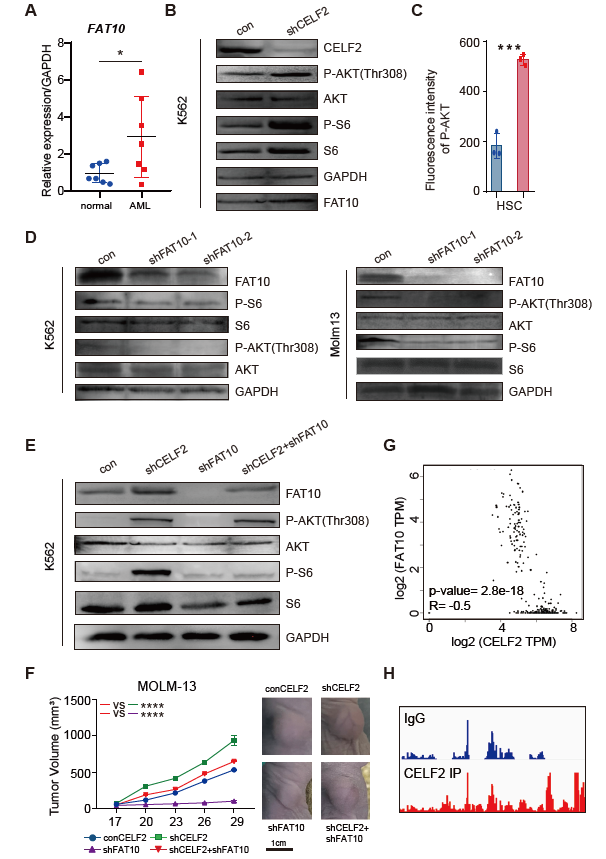
**

Supplemental Figure 5. Loss of CELF2 activates the AKT pathway by regulating the expression of FAT10

(A) Expression of the *CELF2* mRNA in 7 AML patient samples and 7 normal individuals analyzed using RT-PCR. (B) Levels of pathway-related proteins in CELF2 knock-down K562 cells were analyzed by Western blot. (C) Level of P-AKT in *Celf2* KO HSCs and control HSCs. (D) Levels of pathway-related proteins in FAT10 knock-down K562 (left panel) and MOLM-13 (right panel) cells that were analyzed using Western blot. (E) Levels of pathway-related proteins in K562 after transduction with lentivirus expressing control, CELF2-specific shRNA, FAT10-specific shRNA or CELF2-specific shRNA and FAT10-specific shRNA respectively. (F) Knock-down of FAT10 inhibits tumor growth of Molm-13 cells in vivo (left panel) in a subcutaneous tumor model in BALB/c nude mice (right panel). (G) The correlation analysis of CELF2 and FAT10 in AML patients and normal individuals. (H) Integrative Genomics Viewer (IGV) tracks displaying RIP-seq read distributions in FAT10 mRNA.

Supplemental Figure 6


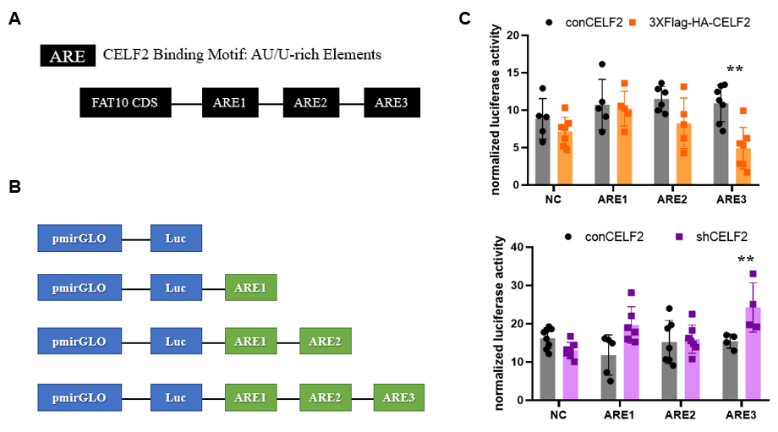


Supplemental Figure 6. CELF2 binds to the AREs in 3'UTR of FAT10.

(A) ARE distribution map of the 3 'UTR region in FAT10 mRNA. (B) Schematic diagram of constructed Luciferase reporters. (C) Determination of Fluc activities in various constructs in K562 cells.

Supplemental Figure 7


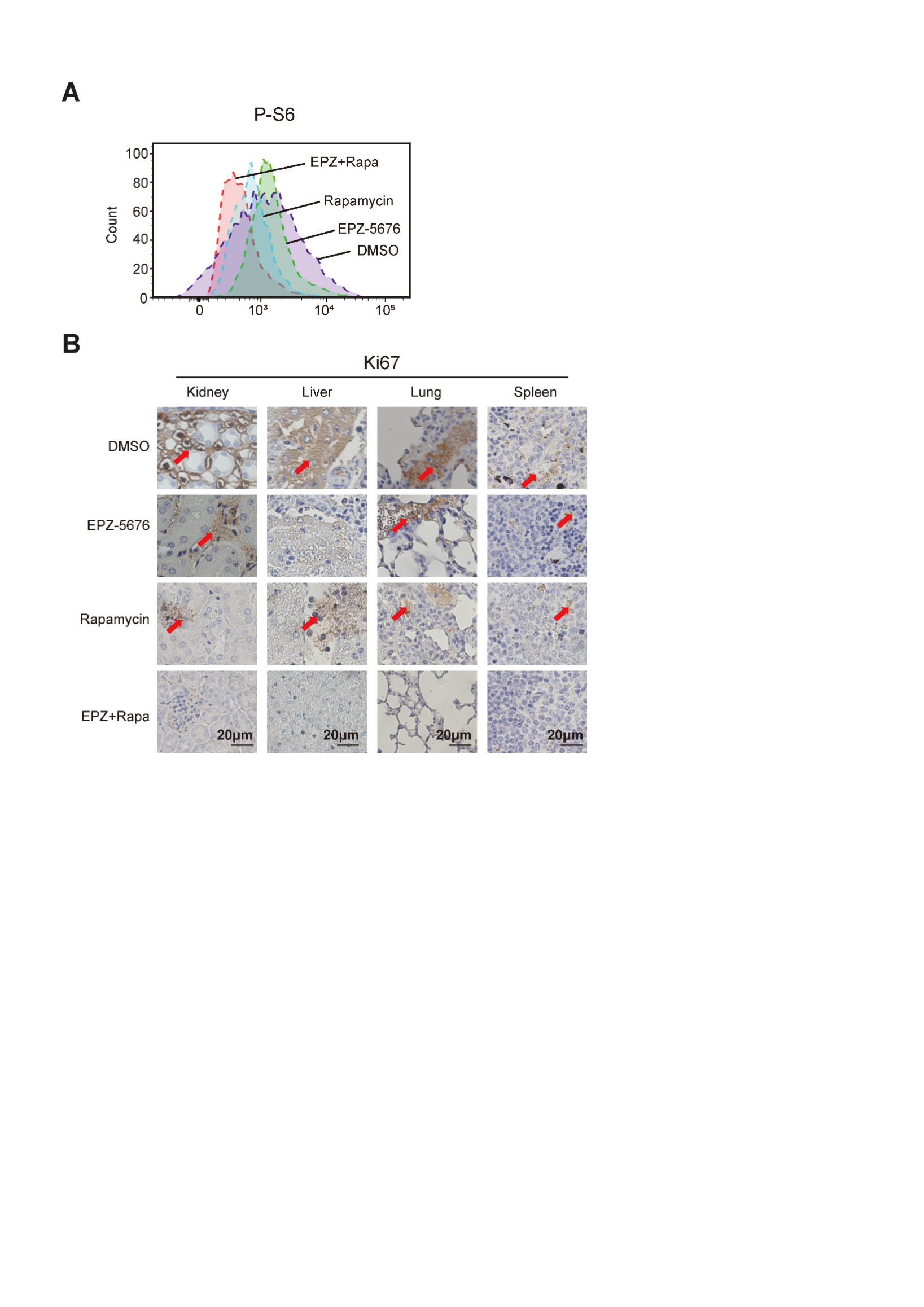


Supplemental Figure 7. Combined treatment with EPZ-5676 and rapamycin prolonged the survival of Celf2 KO+MA9 mice

(A) The phosphorylation level of S6 in BM cells from *Celf2* KO+MA9 mice treated with placebo, EPZ-5676, rapamycin, or combined EPZ-5676 and rapamycin through tail vein injection. (H) Immunohistochemical staining of Ki67 (magnification, 40X) in kidney, liver, lung and spleen from *Celf2* KO+MA9 mice treated with different drugs.

References

1. Yeung YT, Fan S, Lu B, Yin S, Yang S, Nie W, et al. CELF2 suppresses non-small cell lung carcinoma growth by inhibiting the PREX2-PTEN interaction. Carcinogenesis. 2020;41(3):377-89.
